# Change in larval fish assemblage in a USA east coast estuary estimated from twenty-six years of fixed weekly sampling

**DOI:** 10.1371/journal.pone.0224157

**Published:** 2019-10-23

**Authors:** Jason M. Morson, Thomas Grothues, Kenneth W. Able

**Affiliations:** 1 Haskin Shellfish Research Laboratory, Rutgers University, Port Norris, NJ, United States of America; 2 Marine Field Station, Rutgers University, Tuckerton, NJ, United States of America; Australian Bureau of Agricultural and Resource Economics and Sciences, AUSTRALIA

## Abstract

Climate change is leading to significant alterations to ecosystems all over the world and some of the resulting impacts on fish and fisheries are now becoming apparent. Estuaries, which are highly susceptible to climate change because they are relatively shallow and in close proximity to anthropogenic stressors, provide habitat to many fish species at a critical time in the life history, after transport and just prior to settlement in nurseries. Despite this, the long-term impacts of climate change on larval fish at this critical location/stage in the life history are not well documented. The larval fish assemblage of a coastal estuary was sampled once per week for twenty-six years at a fixed location in southern New Jersey, USA. We used ordination and regression analysis to evaluate the whole assemblage, individual species/family occurrence, and trends in total density and diversity over that time. The larval fish assemblage changed significantly in response to warming water temperatures. In addition, approximately one quarter of the species/families in the assemblage exhibited a statistically significant trend in individual occurrence over time. Of these, all five of the five northern-affiliated species decreased in occurrence while 18 of 21 southern-affiliated species increased in occurrence. Finally, total fish density and species diversity increased over the course of the study. The non-uniform response of the species/families in this larval assemblage is similar to what has been documented in other studies that evaluated the temporal trend of open ocean juvenile and adult fish assemblages.

## Introduction

Climate change is a topic of increasing importance because of the current and future implications of its effects, especially in marine and estuarine ecosystems [1– 4]. Increased temperatures, shifting winds, rising water levels, intensified storms and changes in pH and ocean currents [5–7] are only some of the effects of a changing ocean. These effects are leading to significant alterations in coastal ecosystems. For example, numerous works now demonstrate how climate change has impacted fish populations [8–15] and associated fisheries [16–21].

Estuaries, which are important as nurseries for both larval and juvenile stage fish [22–24], are highly susceptible to climate change because they are relatively shallow and thus have low thermal inertia, and generally are in close proximity to anthropogenic stressors [25–28]. Evidence of increasing temperatures and large fluctuations in salinity due to climate change has been documented in estuarine systems in the northern and southern hemispheres [25, 29–32]. This includes along the east coast of the U.S. in large estuaries, like the Hudson River estuary and Narragansett and Delaware Bays, and small ones, such as the Mullica River-Great Bay estuary [23, 33, 34]. Fish larvae that are transported to estuaries may therefore be even more affected by climate change than larger juveniles and adults that have the ability to move to more favorable areas.

Long-term ecological monitoring programs are critical to resolving the issues associated with climate change [e.g., 8, 35–38]. The Rutgers University Marine Field Station (RUMFS) in southern New Jersey has been conducting larval fish sampling weekly at one place in a coastal estuary, behind Little Egg Inlet, under a fixed protocol for 26 years. To date, the assemblage at this site appears similar to adjacent inlets and estuarine thoroughfares and thus is representative of the region [39]. The larval fish assemblage in this estuary was previously described using the first six years of this time series [40]. In addition, it has been compared with samples collected from other estuaries to gauge regional synchrony or to evaluate life history for specific species such as *Anguilla rostrata* [41, 42], *Conger oceanicus* [43], *Paralichthys dentatus* [44, 45], *Brevoortia tyrannus* [46], *Micropogonias undulatus* [47], *and Pseudopleuronectes americanus* [48, 49]. Synthesis of research to date suggests that changes in abundance of selected species are the result of climate change, recovery of local spawning stocks, or other factors that could only be detected with a long time series [23, 47, 49], but no work has yet evaluated the longer-term temporal trends in the entire larval fish assemblage for this data set.

Our specific objective for this paper is to document change in the assemblage of larval fishes in this estuary as estimated from twenty-six years of weekly sampling. Evaluating change at this critical stage in the life cycle and at this critical estuarine location in the coastal ocean system is important for two reasons. First, measuring change at the larval stage addresses distribution at an important, well-defined point in the reproductive cycle because larval abundance is affected by events of the very recent past. The duration of competency for larval settlement is short, typically days. Thus, response signals (fluctuations in larval abundance over time) are less likely to be obfuscated by effects of mixed age, maturity, diet, habitat choice, and other ontological or size correlates expected of a sample of multiple year classes as it would be in fishery-dependent trawl samples, though there is a possibility that these effects could be lagged and thus still manifest in the larval signals. Second, interannual variation in the assemblage of estuarine larvae of open ocean spawners are likely to be more highly correlated with year-class strength than similar metrics calculated from open ocean larval surveys. This is because a large part of the critical phase from spawning to larval settlement, including coastal transport, is complete when larvae arrive in coastal estuaries. This accounts for the influence of, for example, match-mismatch of larvae with predators or food resources at those earlier stages.

## Methods

### Study site

**Fig 1:**
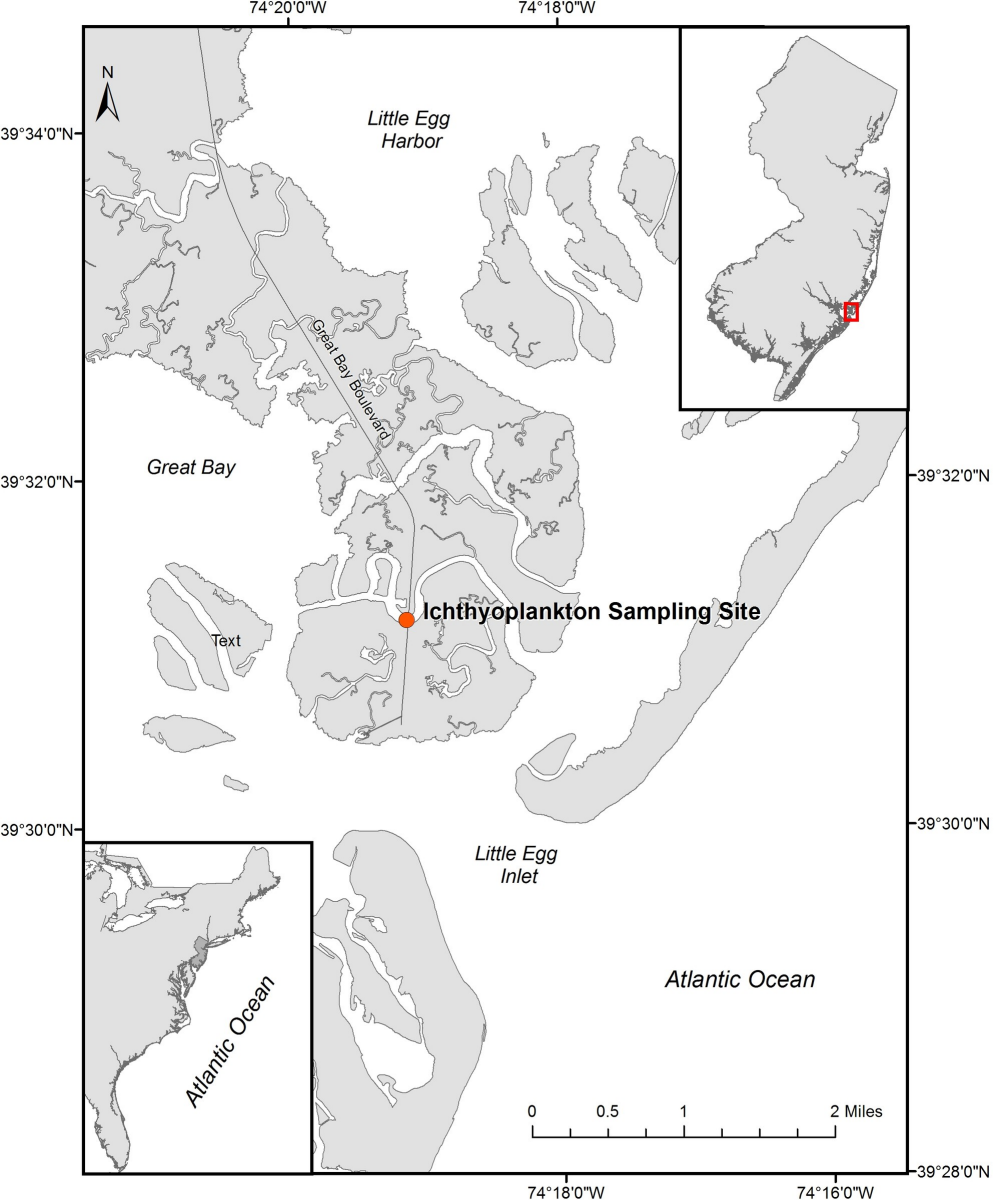
Larval fish sampling location behind Little Egg Inlet in New Jersey (inset).

Larval fish sampling was conducted behind Little Egg Inlet, New Jersey in the northeast United States continental shelf ecosystem (Fig 1). Little Egg Inlet, a relatively unaltered inlet, is the primary source of Atlantic Ocean water that enters two estuaries, a drowned river valley (Mullica River-Great Bay) and an adjacent barrier beach lagoon (Barnegat Bay), and is split by the Sheepshead Meadows peninsula. This dual estuary has a broad, seasonal temperature range (-1.2° to 35° C; [50]), a moderate tidal range, and an average depth of 1.7 m. Sampling was conducted from a bridge over Little Sheepshead Creek (water depth ~3m), a thoroughfare connecting Great Bay and Barnegat Bay across Sheepshead Meadows located 2.5 km from Little Egg Inlet (Fig 1). Atlantic Ocean water flows into the estuary through Little Egg Inlet during flood tides, and portions are diverted into the mouth of Little Sheepshead Creek [48, 51]. Larval fish samples collected from this location are representative of dynamics occurring in the estuary proper [e.g., 31, 40, 48], at other local inlets and thoroughfares [39], as well as the adjacent inner continental shelf [45, 52].

### Sampling methodology

Larval fish entering the estuary were collected with a 1-m diameter, circular plankton net (1-mm mesh). Three 30-minute deployments at 1.5 m water depth during night-time (to reduce gear avoidance) flood tides were made weekly from 1990 to 2015. A General Oceanics flowmeter was attached to the mouth of the net to gauge flow for calculating the water volume sampled for each deployment. Surface water temperature and salinity were recorded at the beginning and end of each sample set. Larval fish were preserved in 95% ethanol and subsequently identified to family or species and counted. Up to 20 individuals from each species for each deployment were selected at random and measured and staged as preflexion, postflexion, or flexion.

### Analytical approach

Larval fish catch data were organized and analyzed at two levels, species and family, and all analyses were repeated and reported for both levels. In addition, the assemblage was evaluated for the subset of data that included those species or families that occurred in at least 1% of the samples over the time series, hereby referred to as commonly occurring, while analyses of individual species or families were performed on the entire data set. Running analyses at both the family and species levels allowed us to make comparisons at these levels as has previously been done for these taxonomically difficult larvae [59].

The mean density (fish/1000 m^3^) of each species or family for each weekly sampling event was calculated from the three replicate tows. The annual mean density for each species was then calculated by averaging the date-specific means. This approach resulted in a year-by-species matrix of annual mean density for each species or family. To identify significant changes in species composition over time, we used this matrix to evaluate change in total larval density and species diversity, change in assemblage for commonly occurring species and families, and change in occurrence for all individual species or families. This multifaceted approach helped to identify recurring patterns in the data at the individual and assemblage levels that are robust to different analytical methods and are therefore likely to indicate true change in the larval fish assemblage. Analyses applied R v3.3.1 statistical computing software [53].

#### Whole assemblage analysis

For commonly occurring species and families, the catch matrices described above were evaluated with principle components analysis (PCA) using the *dudi*.*pca* function in the *ade4* R package [54]. To help equally weight the contribution of low catch and high catch species, data were centered and standardized before the PCA analysis. A statistical approach was used to determine dimensionality for the PCA to reduce the chance of interpreting noise in the data or of missing significant trends [55]. To estimate the total number of principle components to retain for interpretation, we used the *testdim* function in the *ade4* R package [54]. This function calculates the RV coefficient for matrices with alternative dimensions to evaluate whether additional dimensions add relevant information [55]. For each principle component retained, we then regressed the component scores across year and used the *lm* function in the base R package to evaluate whether there was a statistically significant trend over time.

#### Individual/Family-level analysis

Some species or families may occur at any given time in a sample at the tail ends of their environmental niche, and therefore may only be present intermittently when conditions are most favorable. As described previously, these rare (observed in <1% of samples) species or families were removed from the whole assemblage analyses. However, a trend in individual occurrence in samples from a high temporal-resolution, fixed sampling location could indicate a shift in the geographic range minima or maxima for a given species or family. Therefore, we also evaluated the change in presence/absence over time for all species and families. We used a generalized linear model with a logit-link function and a binomial error distribution to evaluate the change in probability of capture over time for each species. Logistic regression models were fit using the standard *glm* function in the base R package [53]. We assigned a binary response (presence/absence) to each species for each sampling event and regressed this response across year. Those species or families that exhibited significant temporal change (p<0.05) were then categorized as declining or increasing in occurrence over time depending on whether the slope of the best fit line was negative or positive, respectively. In addition, some species and families were categorized as either northern (those typically spawning north of Cape Cod, Massachusetts, including Georges Bank) or southern (those typically spawning south of Cape Hatteras, North Carolina) and local (those typically spawning in the Middle Atlantic Bight) based on Able and Fahay [23] and references therein. For this subset of species, we identified how many exhibited statistically significant increasing or declining trends in occurrence.

#### Total density and diversity analysis

To evaluate whether there was a net change in total density or species diversity over time, we first calculated the Shannon and Simpson indices of diversity using the *diversity* function in the *vegan* R package [56]. Total density and each of the diversity indices were then independently regressed across year using the *lm* function in the base R package to evaluate whether there was a statistically significant trend over time [53].

## Results

### Sample description

A total of 1,252 weekly samples were analyzed over the course of twenty-six years from 1990 to 2015. Over 650,000 fish larvae were collected in these samples and identified to family or species. Representative fish from sixty families were observed and forty were observed in at least 1% of the samples (Table 1). A total of 138 different species were identified with sixty-five of those present in at least 1% of the samples (Table 1).

**Table 1:** Scientific and common names, proportion of samples with positive catch, mean overall density, and directional change for species or families, including those from northern, southern, or local (Middle Atlantic Bight) origin (where known), that exhibited significant (p<0.05) change in occurrence over time (blank cells under the Direction of Change column indicate no trend).

Family	Species	Common Name	% of Samples With Positive Catch	Mean Density (fish/ 1000 m^3)	Direction of Change	Source
Achiridae	*Trinectes maculatus*	Hogchoker	<1%	0.001		Local
Albulidae	*Albula vulpes*	Bonefish	<1%	0.001		Southern
Ammodytidae			17%	1.477	Declining	Northern
	*Ammodytes americanus*	American sand lance	<1%	0.001		Northern
	*Ammodytes sp*.	Sand lance	17%	2.952	Declining	Northern
Anguillidae			42%	1.447		Unknown
	*Anguilla rostrata*	American eel	41%	2.891		Southern
	*Anguilliformes sp*.	Eel	<1%	0.001		Unknown
Atherinidae			<1%	0.058		Unknown
	*Atherinidae sp*.	Old world silversides	<1%	0.112		Local
	*Membras martinica*	Rough silverside	<1%	0.004		Southern
Atherinopsidae			51%	4.157	Increasing	Local
	*Menidia beryllina*	Inland silverside	3%	0.075		Local
	*Menidia menidia*	Atlantic silverside	43%	7.520	Increasing	Local
	*Menidia sp*.	Silversides	13%	4.875	Increasing	Local
Batrachoididae	*Opsanus tau*	Oyster toadfish	5%	0.130		Local
Belonidae	*Strongylura marina*	Atlantic needlefish	2%	0.027		Local
Blenniidae			4%	0.025		Southern
	*Blenniidae sp*.	Combtooth blenny	<1%	0.006		Southern
	*Chasmodes bosquianus*	Striped blenny	1%	0.028	Increasing	Southern
	*Hypsoblennius hentz*	Feather blenny	3%	0.040		Southern
Bothidae			17%	0.230	Declining	Unknown
	*Bothus ocellatus*	Eyed flounder	<1%	0.001		Southern
	*Bothus sp*.	Left-eyed flounders	<1%	0.001		Southern
	*Citharichthys spilopterus*	Bay whiff	<1%	0.052		Southern
	*Etropus crossotus*	Fringed flounder	<1%	0.000		Southern
	*Etropus microstomus*	Smallmouth flounder	17%	1.317	Declining	Local
	*Hippoglossina oblonga*	Fourspot flounder	<1%	0.005		Local
Carangidae			<1%	0.001		Southern
	*Caranx hippos*	Crevalle jack	<1%	0.002		Southern
	*Seriola zonata*	Banded rudderfish	<1%	0.001		Southern
Chaetodontidae			<1%	0.001		Southern
	*Chaetodon ocellatus*	Spotfin butterflyfish	<1%	0.000		Southern
	*Chaetodon sp*.	Butterflyfishes	<1%	0.001		Unknown
Clupeidae			70%	7.737	Increasing	Unknown
	*Alosa aestivalis*	Blueback herring	<1%	0.003		Local
	*Alosa mediocris*	Hickory shad	<1%	0.002		Local
	*Brevoortia tyrannus*	Atlantic menhaden	55%	46.068	Increasing	Local
	*Clupea harengus*	Atlantic herring	21%	5.129	Declining	Northern
	*Clupeidae sp*.	Herrings	9%	0.375	Increasing	Unknown
	*Clupeiformes sp*.	Clupeid	13%	2.019	Increasing	Unknown
	*Opisthonema oglinum*	Atlantic thread herring	5%	0.561	Increasing	Southern
Congridae	*Conger oceanicus*	Conger eel	11%	0.423		Southern
Cottidae	*Myoxocephalus aenaeus*	Grubby	4%	0.110		Northern
Cynoglossidae			3%	0.078	Declining	Southern
	*Symphurus plagiusa*	Blackcheek tonguefish	<1%	0.061	Increasing	Southern
	*Symphurus sp*.	Tonguefish	2%	0.095	Declining	Southern
Cyprinodontidae			1%	0.008		
	*Cyprinodon variegatus*	Sheepshead minnow	<1%	0.007		Local
	*Lucania parva*	Rainwater killifish	<1%	0.010	Increasing	Local
Diodontidae			<1%	0.002		Southern
	*Chilomycterus schoepfi*	Striped burrfish	<1%	0.003		Southern
	*Chilomycterus sp*.	burrfish	<1%	0.001		Southern
Eleotridae			1%	0.007	Increasing	Southern
	*Dormitator maculatus*	Fat sleeper	<1%	0.010	Increasing	Southern
	*Erotelis smaragdus*	Emerald sleeper	<1%	0.004	Increasing	Southern
Elopidae			1%	0.007	Increasing	Southern
	*Elops saurus*	Ladyfish	<1%	0.010	Increasing	Southern
	*Megalops atlanticus*	Tarpon	<1%	0.004	Increasing	Southern
Engraulidae			57%	32.233		
	*Anchoa hepsetus*	Striped anchovy	15%	2.973	Increasing	Local
	*Anchoa lyolepis*	Shortfinger anchovy	<1%	0.013		Southern
	*Anchoa mitchilli*	Bay anchovy	55%	150.99		Local
	*Anchoa sp*.	Anchovy	21%	39.236	Increasing	Unknown
	*Engraulidae sp*.	Anchovies	1%	0.136	Increasing	Southern
	*Engraulis eurystole*	Silver anchovy	2%	0.051	Declining	Southern
Fundulidae			15%	0.259	Declining	Local
	*Fundulus heteroclitus*	Mummichog	13%	0.773		Local
	*Fundulus luciae*	Spotfin killifish	<1%	0.001		Local
	*Fundulus majalis*	Striped killifish	3%	0.040		Local
	*Fundulus sp*.	Killifish	2%	0.223		Local
Gadidae			<1%	0.008		
	*Gadus morhua*	Atlantic cod	<1%	0.010		Northern
	*Pollachius virens*	Pollock	<1%	0.005		Northern
Gasterosteidae			15%	0.174		Unknown
	*Apeltes quadracus*	Fourspine stickleback	4%	0.054		Local
	*Gasterosteus aculeatus*	Threespine stickleback	12%	0.294	Declining	Northern
Gerreidae			2%	0.028		Southern
	*Diapterus auratus*	Irish pompano	<1%	0.019		Southern
	*Eucinostomus sp*.	Mojaaras	<1%	0.013		Southern
	*Gerreidae sp*.	Mojaaras	2%	0.051		Southern
Gobiidae			50%	1.919	Increasing	Unknown
	*Ctenogobius boleosoma*	Darter goby	17%	0.671	Increasing	Southern
	*Ctenogobius sp*.	Goby	<1%	0.001		Southern
	*Gobiidae sp*.	Gobies	4%	0.235	Increasing	Unknown
	*Gobionellus oceanicus*	Highfin goby	4%	0.090	Increasing	Southern
	*Gobionellus sp*.	Goby	<1%	0.006		Southern
	*Gobiosoma bosc*	Naked goby	27%	7.894		Local
	*Gobiosoma ginsburgi*	Seaboard goby	23%	6.520	Increasing	Unknown
	*Gobiosoma sp*.	Goby	10%	2.245	Increasing	Unknown
	*Microgobius thalassinus*	Green goby	5%	0.857	Increasing	Southern
Haemulidae			<1%	0.005		Southern
	*Haemulidae sp*.	Grunts	<1%	0.004		Southern
	*Orthopristis chrysoptera*	Pigfish	<1%	0.006		Southern
Hemiramphidae			<1%	0.012	Increasing	Southern
	*Hemiramphus balao*	Balao halfbeak	<1%	0.002		Southern
	*Hyporhamphus meeki*	American halfbeek	<1%	0.017	Increasing	Southern
Labridae			8%	0.379		
	*Tautoga onitis*	Tautog	7%	0.601		Local
	*Tautogolabrus adspersus*	Cunner	3%	0.185		Northern
Lophiidae	*Lophius americanus*	Goosefish	<1%	0.001		Local
Lotidae	*Enchelyopus cimbrius*	Fourbeard rockling	2%	0.061		Northern
Lutjanidae	*Lutjanus griseus*	Gray snapper	<1%	0.003		Southern
Microdesmidae	*Microdesmus longipinnis*	Pink wormfish	<1%	0.006		Southern
Mugilidae			1%	0.003		Unknown
	*Mugil cephalus*	Striped mullet	<1%	0.001		Southern
	*Mugil curema*	White mullet	<1%	0.003		Southern
	*Mugil sp*.	Mullet	<1%	0.006		Unknown
Muraenidae	*Muraenidae sp*.	Moarys	<1%	0.001		Southern
Ophichthidae			8%	0.076		Unknown
	*Myrophis punctatus*	Speckeld worm eel	7%	0.290		Southern
	*Ophichthidae sp*.	Eel	<1%	0.006		Unknown
	*Ophichthus cruentifer*	Margined snake eel	<1%	0.001		Local
	*Ophichthus gomesii*	Shrimp eel	<1%	0.005		Southern
Ophidiidae			4%	0.041	Declining	Unknown
	*Ophidion marginatum*	Striped cusk-eel	2%	0.063		Local
	*Ophidion sp*.	Cusk-eel	1%	0.019	Declining	Unknown
Ostraciidae	*Lactophrys sp*.	Boxfishes	<1%	0.001		Unknown
Paralichthyidae			32%	2.149	Increasing	
	*Paralichthyidae sp*.	Flounder	<1%	0.002		
	*Paralichthys dentatus*	Summer flounder	32%	6.442	Increasing	Local
	*Paralichthys sp*.	Flounder	<1%	0.002		Unknown
Pholidae	*Pholis gunnellus*	Rock gunnel	5%	0.143		Northern
Phycidae			6%	0.031		
	*Urophycis chuss*	Red hake	1%	0.007		Local
	*Urophycis regia*	Spotted hake	4%	0.078		Local
	*Urophycis sp*.	Hake	<1%	0.007		Unknown
Pleuronectidae			19%	10.218		Unknown
	*Limanda ferruginea*	Yellowtail flounder	<1%	0.001		Northern
	*Pseudopleuronectes americanus*	Winter flounder	19%	20.435		Local
Pomatomidae	*Pomatomus saltatrix*	Bluefish	<1%	0.031		Local
Rachycentridae	*Rachycentron canadum*	Cobia	<1%	0.001		Southern
Rajidae	*Raja eglanteria*	Clearnose skate	<1%	0.000		Local
Sciaenidae			48%	2.706	Increasing	Unknown
	*Bairdiella chrysoura*	Silver perch	3%	0.328	Increasing	Southern
	*Cynoscion regalis*	Weakfish	10%	3.525		Local
	*Leiostomus xanthurus*	Spot	5%	0.189		Local
	*Menticirrhus americanus*	Southern kingfish	<1%	0.003		Southern
	*Menticirrhus saxatilis*	Northern kingfish	4%	0.149		Local
	*Menticirrhus sp*.	Kingfish	2%	0.093	Declining	Unknown
	*Micropogonias undulatus*	Atlantic croaker	32%	22.203	Increasing	Southern
	*Pogonias cromis*	Black drum	<1%	0.001		Southern
	*Sciaenidae sp*.	Drums	6%	0.570	Increasing	Unknown
	*Sciaenops ocellatus*	Red drum	<1%	0.001		Southern
Scombridae			1%	0.042		Unknown
	*Scomber scombrus*	Atlantic mackerel	<1%	0.082		Local
	*Scomberomorus maculatus*	Spanish mackerel	<1%	0.001		Southern
Scophthalmidae	*Scophthalmus aquosus*	Windowpane	13%	2.749		Local
Scorpaenidae	*Scorpaena sp*.	Scorpionfish	<1%	0.001		Unknown
Serranidae			6%	0.131	Increasing	Unknown
	*Centropristis striata*	Black sea bass	6%	0.393		Local
	*Mycteroperca microlepis*	Gag	<1%	0.001		Southern
	*Mycteroperca sp*.	Sea basses	<1%	0.001		Southern
Sparidae			2%	0.016	Increasing	Unknown
	*Lagodon rhomboides*	Pinfish	1%	0.014	Increasing	Southern
	*Stenotomus chrysops*	Scup	<1%	0.018		Local
Sphyraenidae	*Sphyraena borealis*	Northern sennet	<1%	0.001		Southern
Stichaeidae			<1%	0.005	Declining	Northern
	*Lumpenus lumpretaeformis*	Snakebelly	<1%	0.000		Northern
	*Ulvaria subbifurcata*	Radiated shanny	<1%	0.009		Northern
Stromateidae			3%	0.059		Unknown
	*Peprilus sp*.	Butterfish	<1%	0.002		Unknown
	*Peprilus triacanthus*	Butterfish	3%	0.115		Local
Syngnathidae			64%	11.115		Local
	*Hippocampus erectus*	Lined seahorse	13%	0.339		Local
	*Syngnathus fuscus*	Northern pipefish	63%	21.887		Local
Synodontidae			2%	0.012		Unknown
	*Synodontidae sp*.	Lizardfishes	<1%	0.000		Southern
	*Synodus foetens*	Inshore lizardfish	2%	0.023		Southern
Tetraodontidae			5%	0.091		Unknown
	*Sphoeroides maculatus*	Northern puffer	5%	0.180		Local
	*Tetraodontidae sp*.	Puffers	<1%	0.001		Unknown
Triglidae			11%	0.104		Unknown
	*Prionotus carolinus*	Northern searobin	4%	0.069		Local
	*Prionotus evolans*	Striped searobin	9%	0.241		Local
	*Prionotus sp*.	Searobin	<1%	0.002		Unknown
Uranoscopidae	*Astroscopus guttatus*	Northern stargazer	4%	0.103		Southern

### Whole assemblage response

Following PCA of the commonly-occurring species, a single component was retained for subsequent analysis, while for the commonly-occurring families analysis, two axes were retained (Table 2). The single axis retained for analysis of the species data accounted for 14% of the variation in the catch, while the two axes retained for analysis of the family data accounted for 14% and 12% of the variation in the catch, respectively (Table 2).

**Table 2:** Eigenvalues, inertia, and cumulative inertia for the first five principle components from the species- and family-level PCA. Components in bold represent those that were kept for subsequent analysis.

	Component	Eigenvalues	Inertia	Cumulative Inertia
**Species**	**1**	**9.178**	**14.12**	**14.12**
Species	2	6.809	10.475	24.59
Species	3	5.813	8.942	33.54
Species	4	4.969	7.644	41.18
Species	5	4.61	7.093	48.27
**Family**	**1**	**5.471**	**14.028**	**14.03**
**Family**	**2**	**4.566**	**11.708**	**25.74**
Family	3	3.394	8.703	34.44
Family	4	3.016	7.734	42.17
Family	5	2.833	7.265	49.44

**Fig 2:**
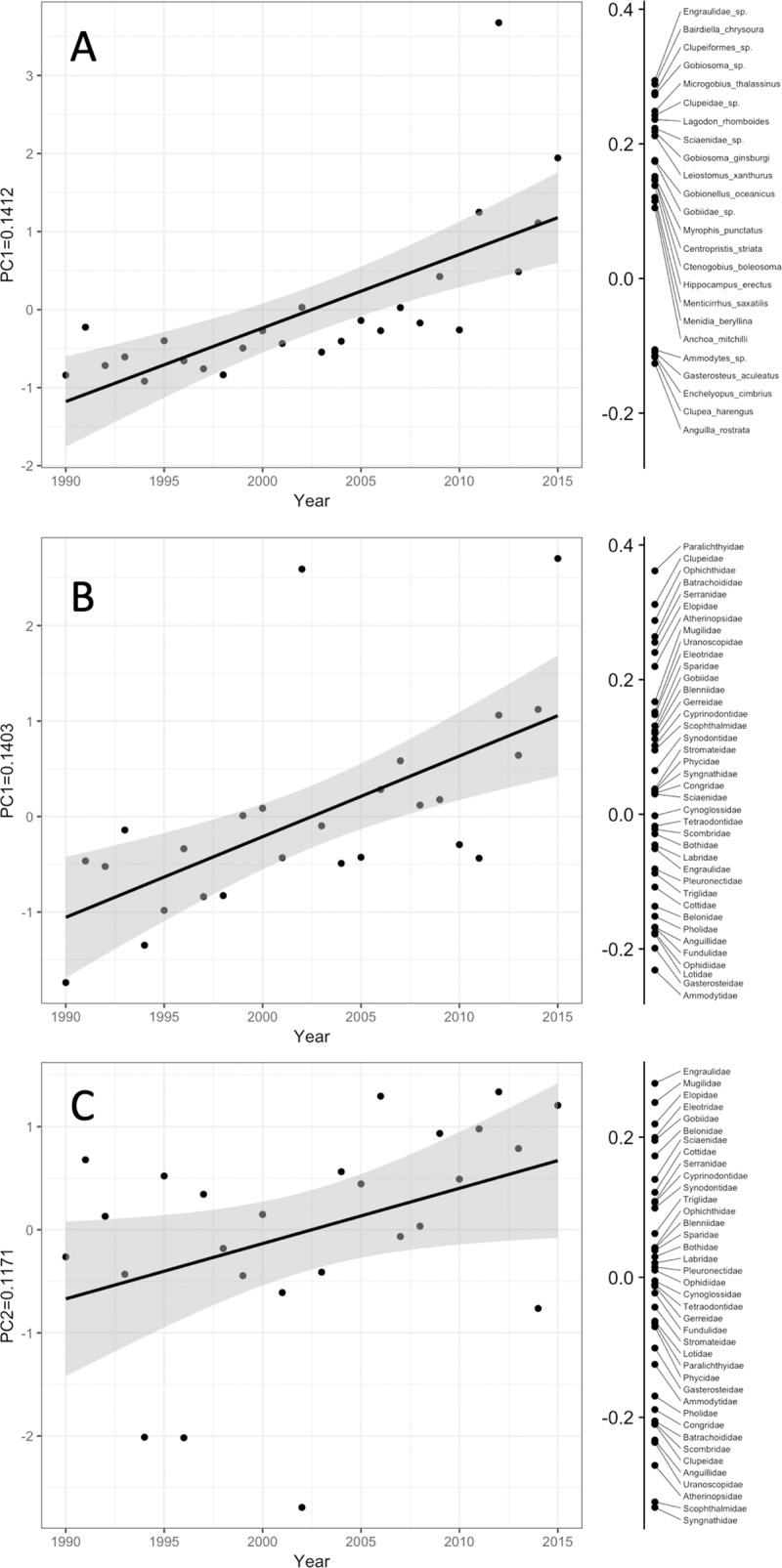
Principle component scores regressed across year (left panels) and species loading scores for the plotted principle component (right panel) for retained principle components from the species-level PCA (A) and the family-level PCA (B, first component and C, second component). *For panel A, all of the species loading scores would not fit vertically on the panel, so only those species scores that weighted heavily on the first principle component are included. See methods for details on selecting the number of principle components to retain.

There was a significant (p<0.0001) annual trend in the first principle component for the species-level PCA (Fig 2A). Some species increased their relative contribution to the assemblage over time. These species, all of which loaded heavily on the first component in the positive direction, included Engraulidae sp., *Bairdella chrysoura*, Clupeiformes sp., *Gobiosoma sp*., *Microgobius thalassinus*, Clupeidae sp., *Lagodon rhomboides*, Sciaenidae sp., *Gobiosoma ginsburgi*, and *Leiostomus xanthurus*. Other species decreased their relative contribution to the assemblage over the course of the time series. These species all loaded heavily on the first component in the negative direction, and included *Anguilla rostrata*, *Clupea harengus*, *Enchelyopus cimbrius*, *Gasterosteus aculeatus*, and *Ammodytes sp*.

For the family-level PCA, both the first (p = 0.0005) and second (p = 0.0417) principle components exhibited significant annual trends (Fig 2B and 2C). For species weighting heavily on the first principle component, paralichthyids, clupeids, ophichthids, batrachoidids, serranids, elopids, and atherinopsids all increased their relative contribution to the assemblage over the time series, while ammodytids, gasterosteids, lotids, ophidiids, fundulids, anguilids, pholids, and belonids decreased their relative contribution (Fig 2B). Of species weighting heavily on the second principle component, engraulids, muglids, eleotrids, and gobiids increased their relative contribution to the assemblage over the time series, while syngnathids, scophthalmids, atherinopsids, uranoscopids, anguillids, and scombrids decreased their relative contribution (Fig 2C).

### Individual/Family-level response

**Fig 3:**
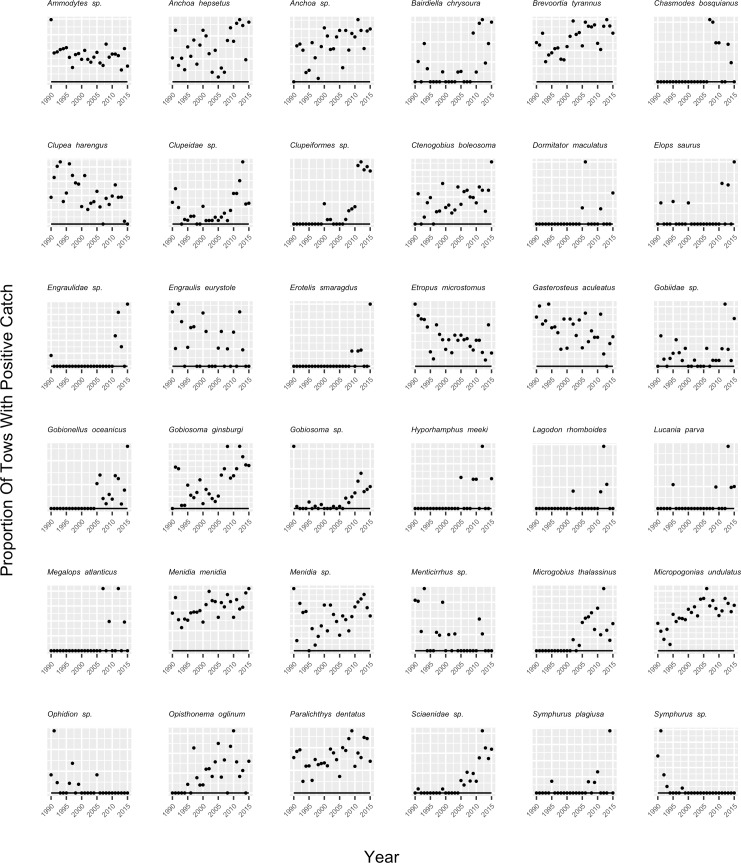
Proportion of tows with positive catch (points) and the probability of a positive catch modeled as a function of year (line) for each species where a significant trend in occurrence was observed. The significance of annual trends in species occurrence were evaluated with logistic regression (see Methods).

**Fig 4:**
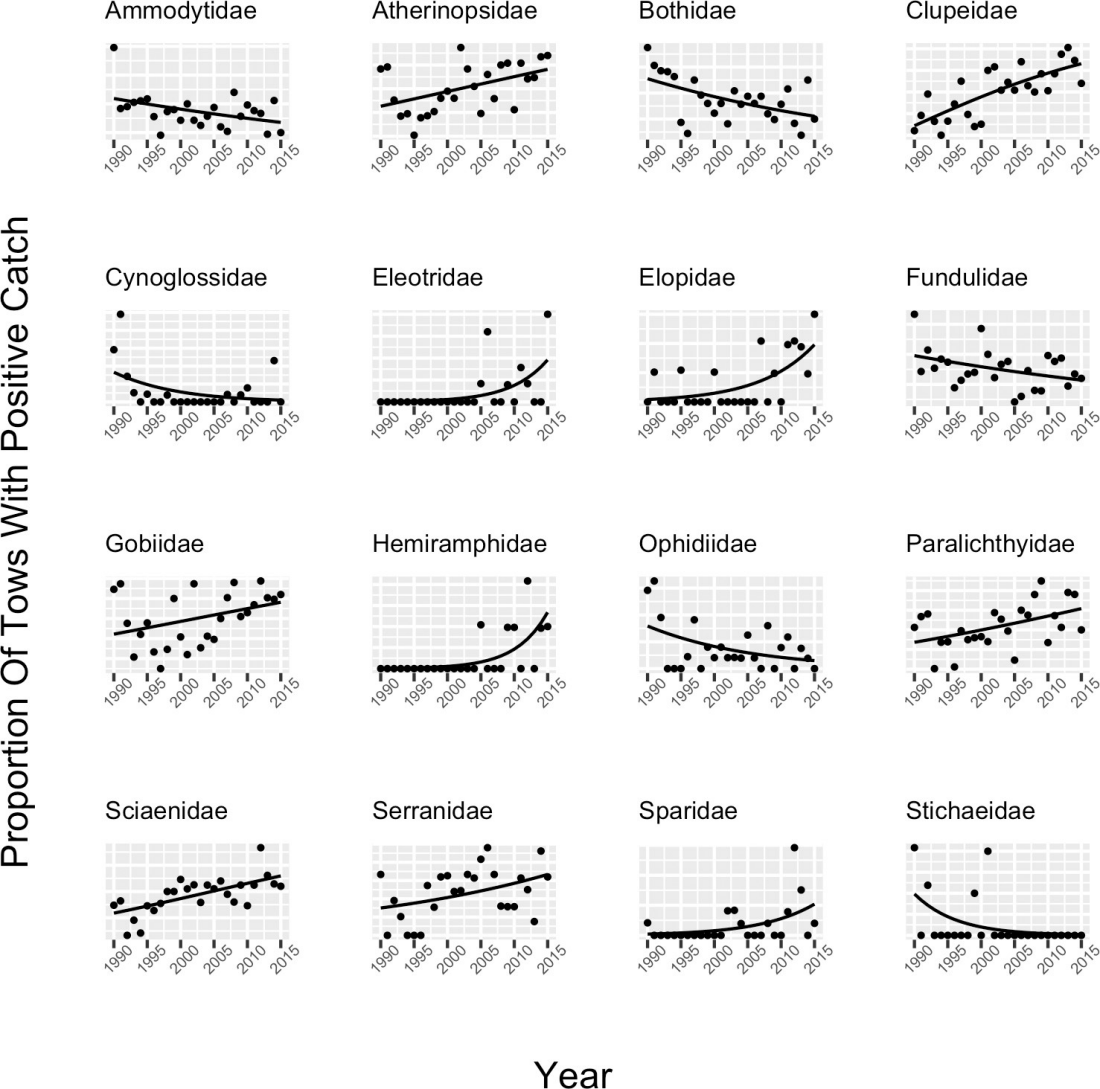
Proportion of tows with positive catch (points) and the probability of a positive catch modeled as a function of year (line) for each family where a significant trend in occurrence was observed. The significance of annual trends in family occurrence were evaluated with logistic regression (see Methods).

Thirty-six (26%) out of 138 species and sixteen (27%) out of sixty families exhibited a statistically significant trend in occurrence over the time series (Table 3, Figs 3 and 4). Of those, ten families increased in occurrence while six declined; twenty-eight species increased in occurrence while only eight declined (Table 3, Figs 3 and 4). While only approximately one quarter of the entire assemblage changed in occurrence, for the commonly-occurring species in the assemblage (those observed in >1% of the samples), twenty-nine (45%) out of sixty-five exhibited a statistically significant trend in occurrence (Table 1, Fig 3).

**Table 3:** Contingency table showing the number and percentage (in parentheses) of families (A) and species (B) from each source declining, increasing, or exhibiting no change in occurrence.

(A)					
Trend	Local	Northern	Southern	Unknown	All
Declining	1 (10%)	2 (40%)	1 (6%)	2 (7%)	6 (10%)
Increasing	1 (10%)	0 (0%)	3 (16%)	6 (23%)	10 (17%)
No Trend	8 (80%)	3 (60%)	14 (78%)	18 (70%)	43 (73%)
(B)					
Trend	Local	Northern	Southern	Unknown	All
Declining	1 (2%)	3 (23%)	2 (3%)	2 (10%)	8 (6%)
Increasing	6 (13%)	0 (0%)	15 (25%)	7 (33%)	28 (20%)
No Trend	38 (85%)	10 (77%)	42 (72%)	12 (57%)	102 (74%)

Some species that showed statistically significant declines in occurrence over time were also identified in the PCA analysis as contributing relatively less over time to the species composition (Figs 2A and 3). These included *Clupea harengus*, *Gasterosteus aculeatus*, and *Ammodytes sp*. Similarly, most species that showed patterns of increasing occurrence were also identified in the PCA analysis as contributing more to the species composition over time, including Engraulidae sp., *B*. *chrysoura*, Clupeiformes sp., *Gobiosoma sp*., *M*. *thalassinus*, Clupeidae sp., *L*. *rhomboides*, Sciaenidae sp., and *G*. *ginsburgi* (Figs 2A and 3).

A similar trend was observed for families that showed significant changes in occurrence. Paralichthyids, clupeids, serranids, elopids, eleotrids, and gobiids both increased in occurrence and increased in their relative contribution to the assemblage, while ammodytids, ophidids, fundulids, and atherinopsids both decreased in occurrence and decreased in their relative contribution to the assemblage (Figs 2B, 2C and 4).

For those species/families that showed significant change in occurrence over time that were also categorized as northern species, five out of five (100%) declined in occurrence, including ammodytids, *Ammodytes sp*., *C*. *harengus*, *G*. *aculeatus*, and stichaeids (Table 3; Figs 3 and 4). Out of twenty-one species/families that showed significant change in occurrence that were also categorized as southern species, eighteen (86%) exhibited increasing occurrence. These included *B*. *chrysoura*, *Chasmodes bosquianus*, *Ctenogobius boleosoma*, *Dormitator maculatus*, eleotrids, elopids, *Elops saurus*, *Engraulidae sp*., *Erotelis smaragdus*, *Gobionellus oceanicus*, *Hyporhamphus meeki*, *L*. *rhomboides*, *Megalops atlanticus*, *M*. *thalassinus*, *M*. *undulatus*, *Opisthonema oglinum*, and *Symphurus plagiusa* (Table 3; Figs 3 and 4).

### Total density and diversity response

**Fig 5:**
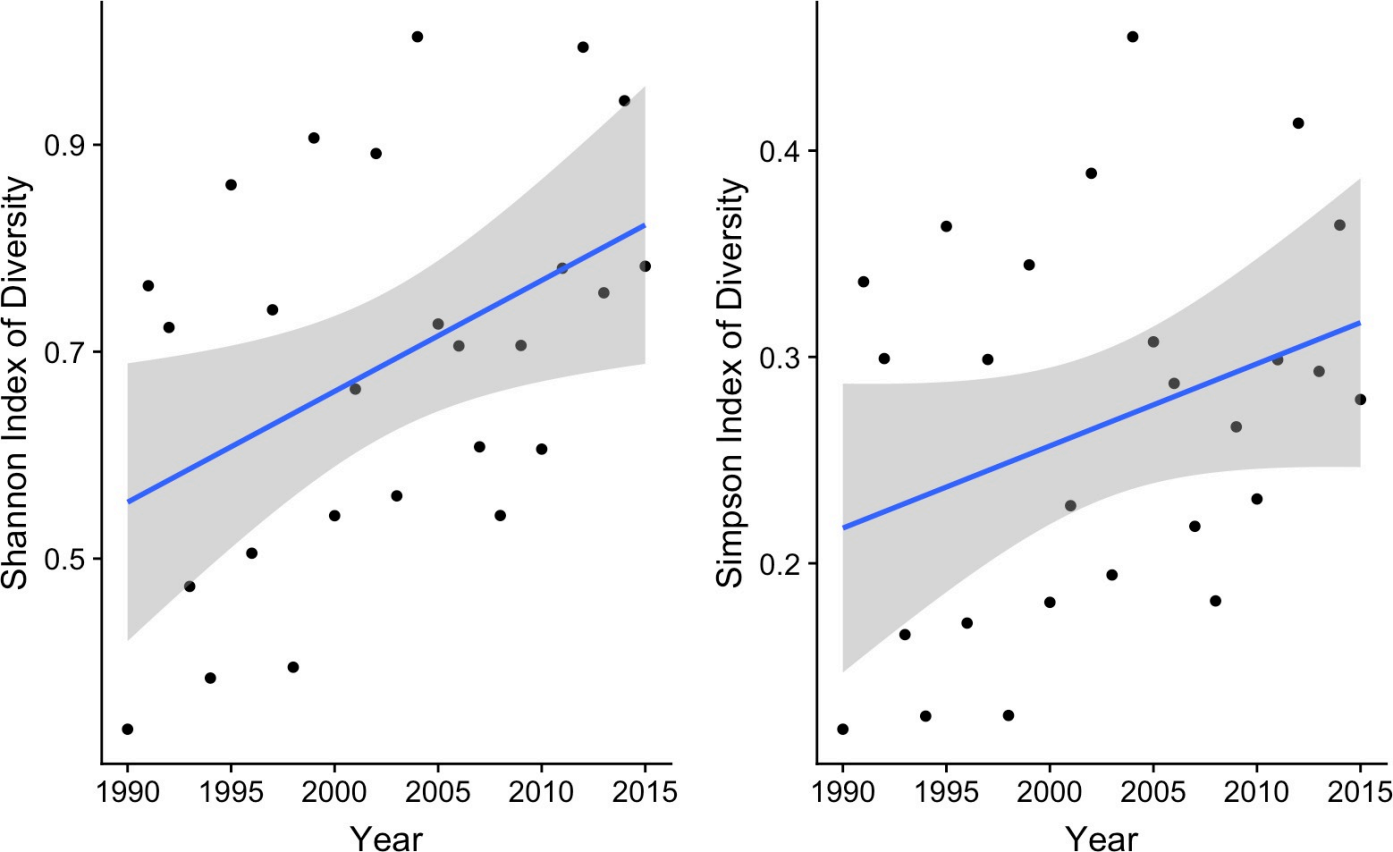
Change in species diversity of the larval fish assemblage at the sampling location over time represented by the Shannon (left panel) and Simpson (right panel) indices of diversity.

**Fig 6:**
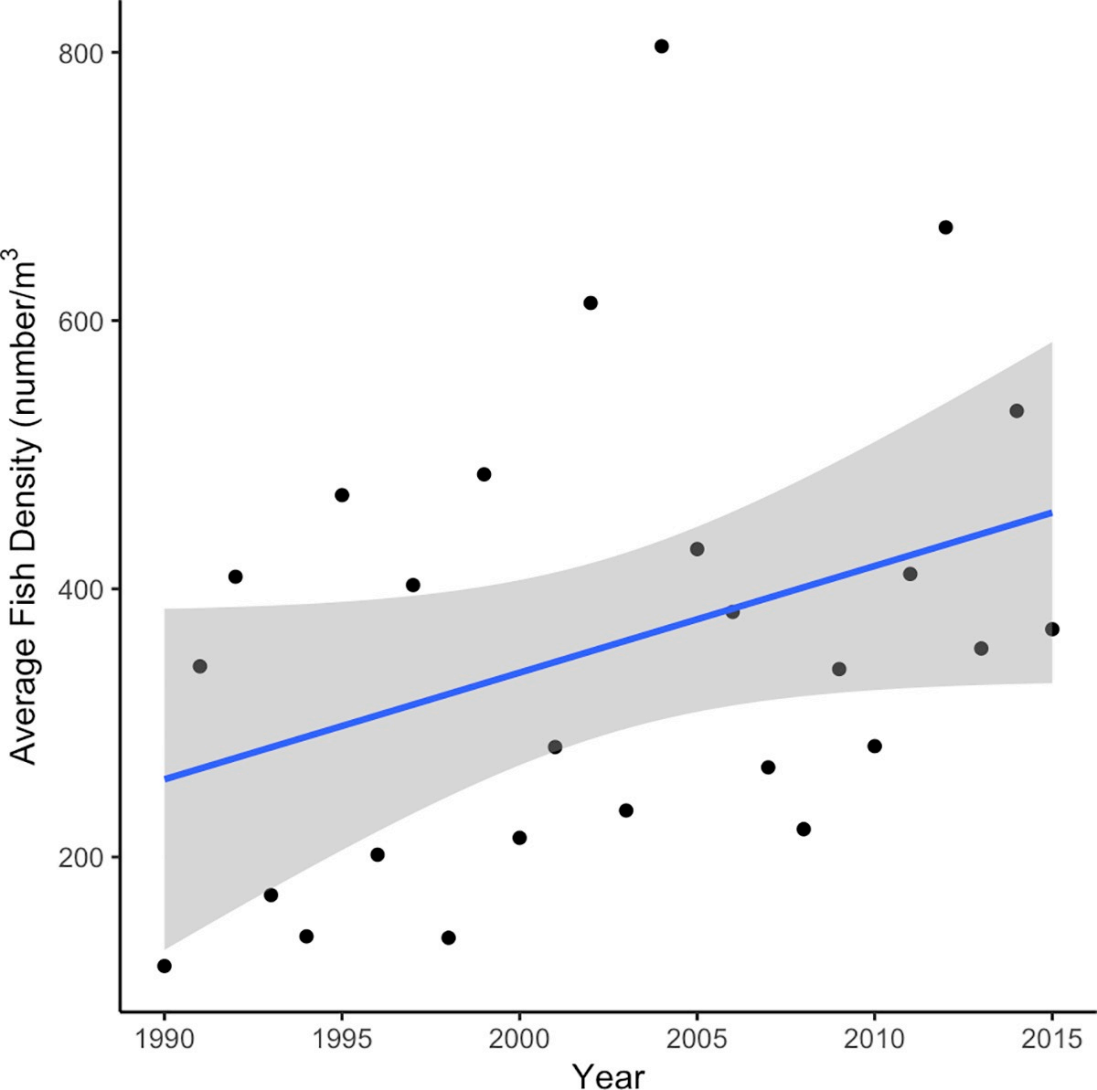
Change in total density of larval fish at the sampling location over time.

Species diversity and total fish density increased over time (Figs 5 and 6). For the Shannon index there was a statistically significant (p = 0.0244) trend at the alpha = 0.05 level, while the Simpson index and total density trends were only significant (p = 0.0998 and p = 0.0783, respectively) at the alpha = 0.10 level.

## Discussion

Long-term monitoring programs are critical to our understanding of how ecosystems are responding to climate change and climate related impacts. Programs that document larval fish assemblages through time, in particular, could serve as early indicators of ecosystem change or of shifting distributions because larval fish occur at low trophic levels [57–59]. Furthermore, documenting change in estuarine larvae from open ocean spawners at the critical phase in development just prior to settlement may provide a more reliable index compared to, for example, open ocean larval fish where larvae have not yet been subjected to coastal transport and associated match-mismatch with resources, predators and prey. However, much of the work documenting the impact of climate change on fish community composition has focused on adult assemblages [e.g. 10, 12, 14]. When larval fish assemblages have been evaluated, the time series have either been short in duration [e.g. 60, 61, 62], focused on larval assemblages from the open ocean [e.g. 4, 58, 63], or when focused on estuarine larvae, evaluated a specific species [e.g. 41, 45, 47] or a selection of species [22, 31]. To our knowledge this is the first time a long-term time series of an entire larval fish assemblage in a coastal estuary has been evaluated for a climate-related trend.

Enhanced warming of the Northwest Atlantic Ocean and Shelf is anticipated to have major consequences for the marine ecosystems there. With this work we identified four significant temporal trends that provide important insight for how larval fish assemblages, for both transient and resident species, in coastal estuaries are responding to rapid environmental change.

First, the overall composition of larval fish at the sampling site changed over time and this was true whether the assemblage was evaluated at the finer, species level or at the coarser, family level. Therefore, this adds to a growing body of literature demonstrating that larval, juvenile, and adult fish communities are responding rapidly to changes in the environment across the world [e.g. 12, 58], including in the Northwest Atlantic Ocean [4, 10, 14, 31]. This approach also helps to address the question of taxonomic sufficiency for difficult larval fishes [59]. We did not attempt to identify the mechanisms controlling the observed change in larval assemblage, partly because the larval fish in this assemblage come from wide ranging origins in the ocean and estuary and are therefore influenced by a range of local and regional conditions that were not measured. However, if we consider the first two PCA axes as climate trends, it suggests 25% of the change in the larval assemblage could be attributed to changing climate. Previous work has demonstrated that bottom temperature can explain more than half of the variation in adult assemblage shifts for species collected in open ocean trawl surveys [12].

Second, when we evaluated change in occurrence at the individual species and family levels we found that only approximately one quarter of the species or families in the larval fish assemblage exhibited statistically significant trends in occurrence and that the significant trends were not unidirectional. Previous evidence has demonstrated that species within an assemblage can have varying responses to changes in the environment [4, 12, 64, 65] and recent work has demonstrated that these responses by fish, as poikilotherms, are likely influenced by a thermal preference specific to each species [66]. Given the diversity of taxa found in the larval fish assemblage evaluated with this work, it is not surprising that some species exhibit no change in occurrence, while others are increasing or decreasing in occurrence. If changes in larval fish assemblage are reliable harbingers of change in juvenile and adult assemblages, these non-uniform responses of individual species within the larval community are likely to alter trophic interactions of adults [67, 68]. However, the overall ecosystem impacts of these changes may be buffered when interacting species are replaced by those with a more tolerant thermal range that also occupy similar trophic guilds [69]. For instance, we observed a decline in the occurrence of *Menticirrhus* spp. and a concurrent increase in the occurrence of *M*. *undulatus*, species at similar trophic levels. Given the changes in the larval fish assemblage we observed, future efforts could focus on modeling how these changes are likely to impact interactions between species in this assemblage or in their subsequent recruits and what those changing interactions suggest about supply to nursery habitats.

Third, for nearly every species or family that exhibited a significant trend in occurrence that was also categorized as either southern or northern relative to the fixed sampling location, the trend was as expected given warming conditions [70] and given previous evidence that larvae and adult fish in this region are shifting predominately northward [4, 12]. That is, northern species, or those likely to have a thermal range skewed toward colder water temperatures, declined in occurrence, and southern species, or those likely to have a thermal range skewed toward warmer water, increased in occurrence, over the same time frame that the region experienced rapid warming. A similar trend was found when a shorter section of this time series was evaluated for a subset of species in the assemblage [31].

Fourth, over 70% of the species that exhibited significant change over the time series increased in occurrence, resulting in an increase in total diversity, and likely total abundance. This is most obvious for the larvae of species that occurred for the first time late in the time series and were consistently present thereafter. For example, *C*. *bosquianus* was not observed in the assemblage from 1990 to 2006, but was observed for six of the following nine years. Similarly, *G*. *oceanicus* was not observed from 1990 to 2004, but was observed every year after (2005–2015). As a result, these species have likely become residents of this estuary over the duration (twenty-six years) of the times series we evaluated. A trend of increasing diversity is supported by previous work [71], including for juvenile and adult fishes in the same region [12, 66], and suggests either a range expansion or a range shift is occurring for some species. When trends in marine species richness were evaluated for trawl data collected from nine different regions around the world (Gulf of Mexico, Northeast US, Eastern Bering Sea, Southeast US, Gulf of Alaska, West Coast US, Newfoundland, Aleutian Islands, and Scotian Shelf), eight showed positive trends and four had statistically significant trends [72]. The authors offer that an expansion in the range of transient species over time could be causing a net increase in richness.

The impact of shifts in fish distribution, like the ones documented here, extends beyond the ecological consequences for the larval community and the subsequent recruits and adult fish. Shifts in marine species distributions also present a challenging set of obstacles for how fisheries are managed because management has traditionally relied on political boundaries for surveying populations, assessing stocks, and allocating quota [20, 21, 73]. These obstacles will require unique and innovative solutions that take into account alternative management scenarios under different climate projections [74, 75]. Time series of larval fish assemblages like this work could prove useful in this endeavor because observing and understanding the delivery of larval fishes to estuaries, both in terms of abundance and timing, provides an opportunity for predicting change [76]. Efforts are already underway to predict changes in distribution of marine species using habitat projection models [e.g. 66, 77], however these models can be highly uncertain [70]. Perhaps data collected from larval fish time series could be used in tandem with habitat projection models to better predict shifts in distributions of juveniles and adults before they happen, and this knowledge could serve as a tool for fishery managers to develop proactive solutions to management problems before they occur.
