# Correction: Change in larval fish assemblage in a USA east coast estuary estimated from twenty-six years of fixed weekly sampling

**DOI:** 10.1371/journal.pone.0225526

**Published:** 2019-11-14

**Authors:** Jason M. Morson, Thomas Grothues, Kenneth W. Able

Fig 3, “Proportion of tows with positive catch (points) and the probability of a positive catch modeled as a function of year (line) for each species where a significant trend in occurrence was observed,” is missing trend lines. Please see the correct Fig 3 here.

**Fig 3:**
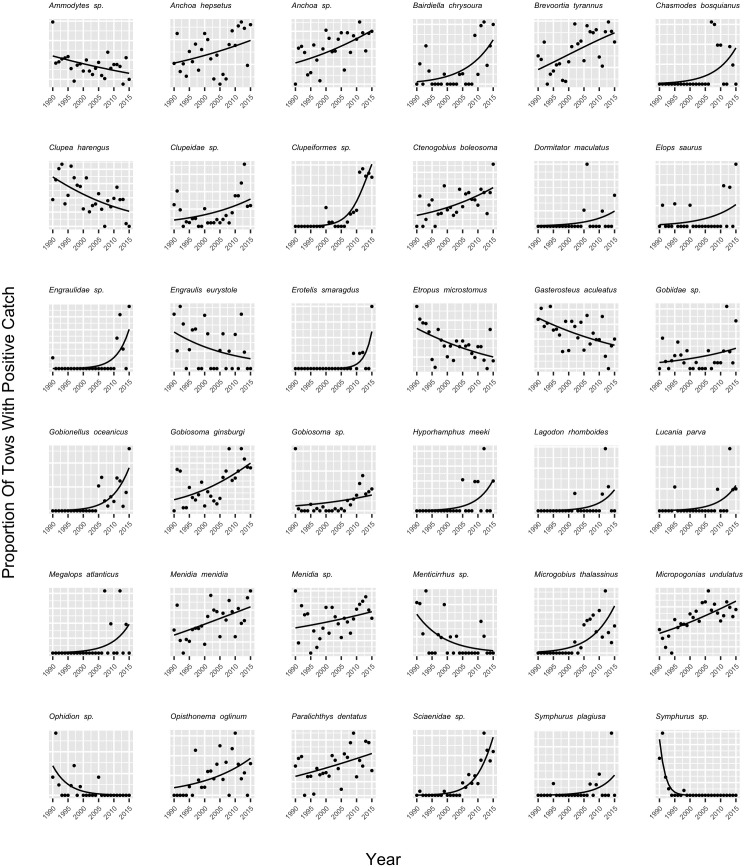
Proportion of tows with positive catch (points) and the probability of a positive catch modeled as a function of year (line) for each species where a significant trend in occurrence was observed. The significance of annual trends in species occurrence were evaluated with logistic regression (see Methods).
